# 

**Published:** 1894-11

**Authors:** 


### CONTENTS FOR NOVEMBER, 1894.

ORIGINAL ADDRESSES. PAGE.

A Review of the Relations Between the State and the Medical Profession in New York. By FRANK WHITEHILL HINKEL, A. M., M. D. .......... 193
On the Clinical and Bacteriological Aspect of Mixed or Concurrent Infection in Pulmonary Tuberculosis. By HENRY L. ELSNER, M. D .......... 204

ORIGINAL COMMUNICATION.

Ambulatory Treatment in Fractures of the Lower Extremities. By M. HARTWIG, M. D. 221

SOCIETY PROCEEDINGS.

Transactions American Association of Obstetricians and Gynecologists .......... 223
The Medical Association of Central New York .......... 242
Buffalo Academy of Medicine .......... 247

EDITORIAL.

Oliver Wendell Holmes .......... 249
Professor Helmholtz .......... 251
State Examinations for License .......... 253

Personal .......... 254
Society Meetings .......... 254
Medical College Notes .......... 248
Books Received .......... 256

